# The Need of Maternity Homes

**Published:** 1922-07

**Authors:** 


### THE NEED OF MATERNITY HOMES.

**(Communicated.)**

THE National Association for the Prevention of Infant Mortality and the National Baby Week Council are holding a series of conferences on infant welfare on July 3, 4 and 5. The importance of these conferences is emphasised by the need for maintaining and improving the present low rate of infant mortality, which last year increased from 80 to 83 per 1,000. As is well known, during the last fifteen years the rate has steadily decreased, from 128 in 1905, for example, to the lowest rate on record in 1920, of 80. The increase last year, though slight, points to the necessity for incessant watchfulness and care, especially during hot summers.

But even more disquieting is the recent increase in the number of maternal deaths due to pregnancy and child-bearing. In 1920 there were 3,942 deaths, representing a death-rate of 4.12. In 1919 the puerperal mortality was higher than in any year since 1905, and the increase was preponderantly due to sepsis. Last year (1921) the maternal mortality rate fell, but is still far higher than it should be, considering our modern knowledge of the cause and prevention of infection and the successful application of this knowledge to the practice of surgery.

In order to arrest this regrettable and unnecessary loss of maternal lives and damaged health of those who survive, there are three main lines of defence: first, a well-developed system of ante-natal care and supervision; secondly, a skilled and careful midwifery service, together with a supply of sterilised equipment and appliances, is needed in the homes of the people; thirdly, the accommodation in maternity homes and hospitals for mothers, for cases when serious difficulties are anticipated, when watchfulness is necessary, when private homes are unsatisfactory, or for unforeseen emergencies, needs to be increased.

Maternity homes are at present in a relatively experimental stage. The facts contained in this article have been largely provided by those who have had actual experience of practical needs. On the whole, small maternity homes of, say, ten to twenty beds for normal or slightly abnormal cases are more needed than hospitals for larger numbers. There are existing maternity hospitals which deal with many of the seriously abnormal cases. A small home where skilled attendance and surgical cleanliness may be found can be simply and economically fitted up by many local authorities. Further, maternity homes should be as accessible as possible, and they are needed as much in small towns and country districts as in large county boroughs. In Wiltshire, for example, arrangements have been made for five small centres, connected with existing homes or hospitals, at Salisbury, Malmesbury, Swindon, Corsham and Calne. The Ministry of Health has now recognised between 90 and 100 maternity homes in England and Wales, providing about 1,034 beds.

Few of these homes are in modern buildings. Where there is money available for building, the "Memorandum in Regard to Maternity Homes and Hospitals, with Plans," issued by the Ministry in January, 1920 (M.C.W.15), is useful. It is much more difficult, however, to adapt satisfactorily and cheaply the large mansion which is usually suggested as an ideal maternity home. The Memorandum above referred to rightly insists upon the necessity of separation accommodation for cases suspected of infection, for adequate staff quarters, and for sufficient sanitary accommodation. Certain maternity homes fail because of the lack of these essentials.

Another difficulty is the comparatively high cost of maintenance, though as the cost of living falls this should become less. Undue economies in staffing or equipment easily lead to disaster. A most capable nurse is needed as matron, and yet in the small homes the salary may not be high enough, or the opportunities sufficiently great, to attract a woman competent to bear the heavy responsibilities involved.

There are also the difficulties of providing suitably for the different types of patient needing care. A pre-maternity ward is most desirable. Difficult questions arise regarding patients suffering from tuberculosis or from other diseases, or as to unmarried girls. Provision ought to be made for the middle-class woman. The wife of the ex-officer, for example, may find it far more difficult to leave her family than the woman who lives in some poor district where neighbours are ready to come in and help. In recent years the needs of the mothers of what may be described as the black-coated classes have too often been forgotten.

Fortunately, some of the new maternity homes are recognising these needs. It is impossible in a short article to convey all the features of the ideal maternity home. Convenient accommodation, modern equipment and a competent staff are all essential, but are not enough by themselves. "The right spirit and atmosphere are indispensable if the home is to become a source of encouragement to motherhood, an example of healthy, contented maternity and a haven of hope and fortitude."

A visit to a well-organised home will teach far more than columns of print. The Queen Mary's Maternity Home at Hampstead, five minutes from the Tube station, is a model and pioneer institution. St. Mary's Hospital at Croydon, under voluntary management, provides accommodation for 17 patients in a large house, standing in a pleasant garden. The Municipal Maternity Homes in Lewisham, Hammersmith, Fulham, Battersea and Wandsworth, to mention a few in the London area, are also good examples of proper organisation. And there is, fortunately, an increasing number in various parts of the country.

The maternity home is one link in the chain related on the one hand to the ante-natal and post-natal clinic, and on the other to the medical school and its consulting staff. It is one of the most effective weapons in the present struggle to raise the standard of midwifery. For this reason it is an institution of the highest national importance.

